# Supplementary material for: Radiological and Clinical Efficacy of Intra-Arterial 90Y-DOTATATE in Patients with Unresectable, Progressive, Liver Dominant Neuroendocrine Neoplasms
Source: J Clin Med. 2021 Apr 20;10(8):1794. doi: 10.3390/jcm10081794 (PMC8074370; doi:10.3390/jcm10081794)
Supplement: Supplementary file 1 [file jcm-10-01794-s001.zip › jcm-1165795-supplementary.pdf]

Preparation of Radiotracer

Labeling: 100 μg DOTA-D-Phe^{1}-Tyr^{3}-octreotate was labeled with max. 7.5 GBq ^{90}Y in no more than 0.5 ml in 0.05 M HCl (POLATOM, Poland) incubating at 95 °C for 25 min. In all chromatography runs, radiochemical purity, which was > 99.0%, with no need for purification Immediately before therapy, the ^{90}Y-DOTATATE in mean activity per session was 1.15 GBq. It was diluted in saline to a final volume of 10 ml. The whole proceedings of labeling are mentioned previously [9].

Radiology Imaging-CT

CT examination of the abdomen and pelvis was performed after i.v. contrast enhancement, in each case approx. 70-120 ml (1.4 mg/kg) of low ionic contrast medium intravenously, at a rate of 3.5-5.2 ml/s. Arterial phase of angioCT of the abdomen and pelvis was followed by the portal venous phase (expanded usually through the chest and then abdomen and pelvis) and the equilibrium phase. The time of the acquisition after i.v. contrast enhancement was as follows: 30, 60, and 240 s, respectively. Contiguously reconstructed sections (pitch of 1:1): Each spiral CT acquisition through the body was accomplished during a breath-hold moment. A standard 512×512 matrix was used in each case. The slice thickness was 1 mm, which was then used to produce reformatting images using the MIP and MPR techniques and 3D reconstruction. A transverse coronal and sagittal projection were used in every case, considering image analysis. CT images were interpreted using a dedicated workstation with total freedom for window and level adjustments, and for the magnification of each image at the time of the analysis.

MRI

Each MRI study was performed on a 1.5-T system with a SENSE phased array abdominal body coil. Images of the liver were acquired using breath-hold or respiratory-triggered techniques (with a navigator-echo technique). In each case, FSE T2 w images were used as a standard approach to detect liver metastases. All parameters of selected sequences of MR examination, including FSE T2 wi fat suppressed, Fast FE T1 wi (Ultrafast Gradient Echo Sequence) after i.v. standard Gd-DTPA injection, and also tested fat suppressed, single shot SE EP DWI performed before i.v. contrast injection.

Dynamic MRI using FFE T1 w images were acquired after i.v. contrast enhancement with (Gd-DTPA) at a dose of 0.2 mmol/kg, administered as a bolus (2–3 ml/s) followed by a 20-mL saline flush (2 mL/sec). At least three time points were used, centred on the arterial, portal venous, and late phases. MR images were acquired 20 and 60 s after contrast injection and equilibrium after 3 min; additionally, a late phase of (Gd-DTPA) images were acquired at least 20 minutes after contrast agent i.v. administration.

SPECT Imaging

"Bremsstrahlung" images were acquired between eight hours and 18 hours after administration of ^{90}Y-DOTATATE using a dual-head gamma camera (e-cam; Siemens) equipped with medium energy collimation with a photopeak centered on 95 keV with a 50% window. The WB images were acquired in each case using standard matrix 1028x256,

with table speed 10 cm/min. The scan was performed to determine presence and localisation of injected activity; also, in each case, SPECT images were acquired, which include whole liver and abdomen with pelvis. Routinely, we used 64 projections (a 128x128 matrix), 24 s per projection with no zoom. Reconstruction algorithms were based on commercially available iterative reconstruction software, i.e., OSEM (3D flash), including four subsets and four iterations with a standard Gaussian filter (3D flash; e-soft Workstation version 6.5, Siemens, USA).
